# Supplementary material for: BestCRM: An Exhaustive Search for Optimal Cis-Regulatory Modules in Promoters Accelerated by the Multidimensional Hash Function
Source: Int J Mol Sci. 2024 Feb 5;25(3):1903. doi: 10.3390/ijms25031903 (PMC10856692; doi:10.3390/ijms25031903)
Supplement: Supplementary file 1 [file ijms-25-01903-s001.zip › ijms-2778462-supplementary.pdf]

Supplementary information for the article: “BestCRM: exhaustive search for optimal Cis-Regulatory Modules in promoters accelerated by multidimensional hash function”.

Igor V. Deyneko

Representation of a CRM

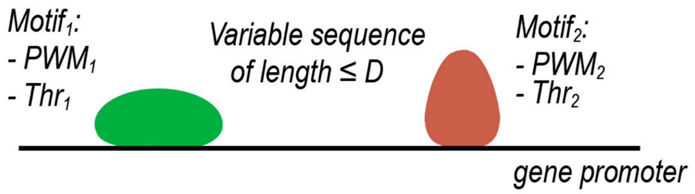

Figure S1. Representation of cis-regulatory module. CRM consists of two DNA motifs, identified by position weigth matrices with respective thresholds (minimal PWM scores) and maximal distance between the motifs.

Building the hash function

For each pair of TFs:

- Find single motifs in both datasets for threshold values (1.0 – 0.3). 0.3 is used as a sufficiently low threshold, since for all PWMs the threshold 0.3 yields approximately one motif per 25 nucleotides on average.
- build the hash function for positive and negative sets.

- define aa area where boundary conditions on minimal and maximal coverage of positive and negative sets are fulfilled.
- Maximize the ratio C^+_{CRM}/C^-_{CRM} within the defined parameters area.
- Output CRM if $\max(C^+_{CRM}/C^-_{CRM})$ is above the value defined by user (default 1.5)

The hash function

The major part of the algorithm is the building of a hash function of distribution of motif pairs on positive and negative promoters. The idea is to make such a function, which shows the portion of sequences in a set that have at least one module depending on PWM thresholds and distance in-between. Mathematically it can be defined as follows.

First we need an indicator function:

$$I(seq,x,y,D)=\begin{cases}1, iff\ \exists(m_1,m_2)\,|\,m_1,m_2\in seq,\ PWM_1(m_1)\geq x, PWM_2(m_2)\geq y, |(m_1,m_2)|\leq D,\\ 0, otherwise.\end{cases},$$

where *seq* is a DNA sequence, *x*, *y* – thresholds for PWM_1, PWM_2, m_1, m_2 – DNA motifs, *L* – maximum distance between motifs.

Then, the hash function will be:

$$H(x,y)=\sum_{seq=1\ldots N} I(seq,x,y)/N,$$

where *N* is a number of DNA sequences in a set. This way $H^+(x,y)$ and $H^-(x,y)$ is defined for positive and negative promoter sets.

This function shows the portion of sequences that contain at least one pair of motifs m_1, m_2 defined by position weight matrices PWM_1, PWM_2 with scores higher than *x*, *y* respectively. The distance between the motifs is restricted by value *D*.

One important property of the hash function is its continuous grow from zero to one along *x*, *y* and *D* axes. Indeed, if the modules is found for (x,y,D), than it will be found also for any scores

lower than x, y, or distances higher than D. In other words, the number of modules will only grow with the decrease of scores and an increase of the allowed distance. This property is used for an efficient implementation of the search strategy.

On the figure S2 there is another example for factors SpoOA and PerR from Prodoric database.

Figure S2. The hash functions $H^{+}(x,y)$ (green) and $H^{-}(x,y)$ (blue) on positive and negative set of promoters respectively. Grey planes indicate default boundary conditions C_{min}=0.75 (upper) and C_{max}=0.5 (lower). Used PWM matrices are SpoOA and PerR from Prodoric database. The area where $H^{+}(x,y)$ and $H^{-}(x,y)$ are above and below respective boundary conditions is marked in red on both grey planes and as a projection on the bottom (solid red). It represents the area of possible solutions and covers only ~3% of the initial parameter variation space.

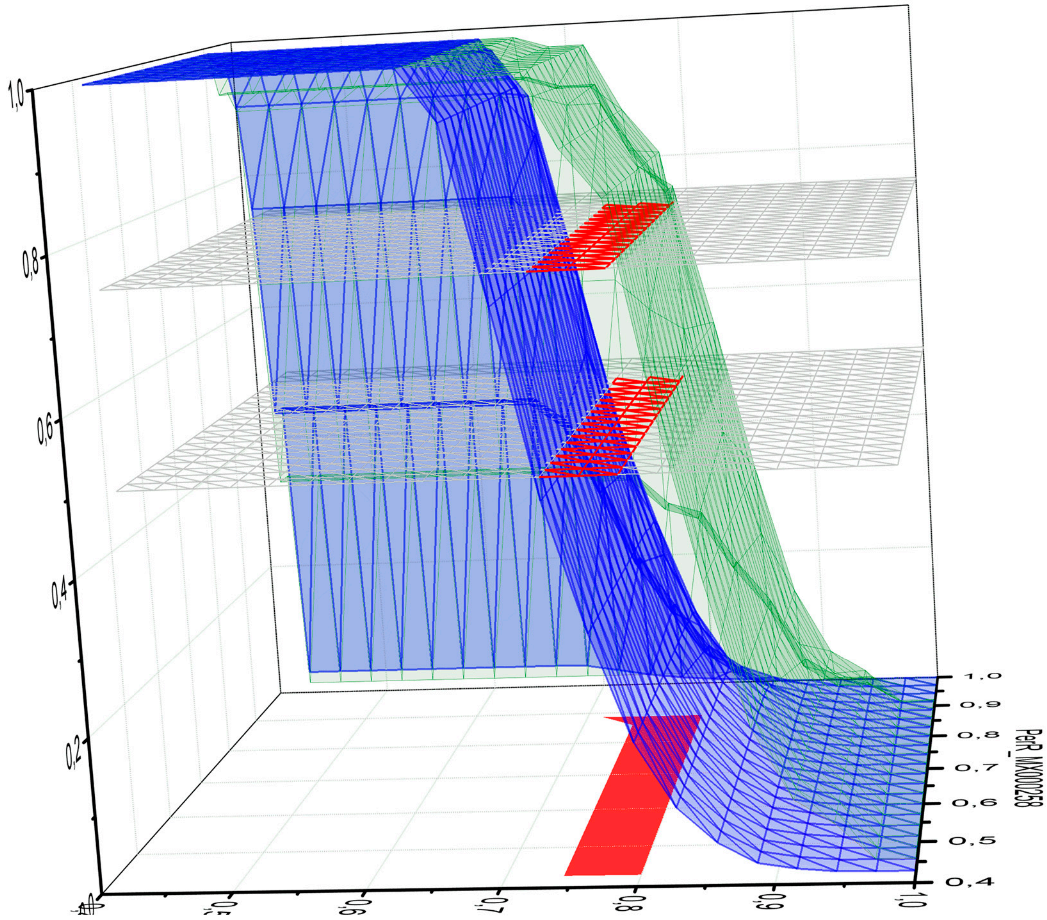

Maximization procedure

The function, which shows the preference of a motif pair for positive set against the negative is given by the ratio:

$$H^{+}(x,y,D) \Big/ H^{-}(x,y,D) \rightarrow \max_{0\le x,y\le 1} \text{ , giving that } H^{+}(x,y,D) \ge C_{\min}{}^{+}, H^{-}(x,y,D) \le C_{\max}{}^{-}$$

An exhaustive search against all possible combinations of PWM thresholds and distance D is optimized via significant reduction of the search space, which is done in several steps. Here we simplify the explanation by considering only PWM thresholds.

The idea behind the first step is to locate a square in (x,y) plane where coverage of the positive set is equal or higher than the required minimum. This can be done with linear complexity by checking values along x axis and finding the minimum x^+ so that $H^+(x^+,1) \ge C_{min}$. Similarly the program finds minimum y^+, so that $H^+(1,y^+) \ge C_{min}$. As a result the possible solution space can be reduced to the square defined by the diagonal $[(x^{+,} y^{+});(1,1)]$.

The same way a square with a diagonal $[(0,0);(x^{-,} y^{-})]$ is defined for negative set, where $H^-(x^-,0) \le C_{max}$ and $H^-(0,y^-) \le C_{max}$. Since we require that both boundary conditions are met the final search space is reduce to the square with diagonal $[(x^+ y^+);(x^{-,} y^-)]$. In reality, the actual area is even smaller, since it may have more complex shape inside the defined square

As it is evident from the figure S2, the suggested optimization procedure significantly reduces the search solutions area. In the presented example, it is only ~3% from the entire square (0...1;0...1).

Of note, if either $x^- < x^+$ or $y^- < y^+$, than there is no possible solution for this motif pair. In other words, this particular module is too frequent on negative sequences and/or too rare on the positive.

At the second step, the search space can be further reduced by recursive checking of the adjacent points starting from one of the boundary values $(x^+,1)$ or $(1, y^+)$ for the positive dataset. So that if a point (x_i, y_i) fulfils the criteria $H^+(x_i, y_i) \ge C+_{min}$, than it is required to check two adjacent points:

$$(x_i, y_i) \rightarrow \begin{cases} if \quad H^{+}(x_i + \Delta x, y_i) & \ge C_{\min} \\ if \quad H^{+}(x_i, y_i + \Delta y) & \ge C_{\min} \end{cases}.$$

Once the criteria are met the program recursively continues with the new point. Similarly, it is done for the negative set of DNA sequences. This forms an accurate shape of parameters where optimization should be performed. In our example on Figure S2, this area (solid red) occupies only ~3% of the initial search space.

Maximization of the ratio $H^{+}(x,y,D) \big/ H^{-}(x,y,D) \to \max_{0 \le x,y \le 1}$ is done by the exhaustive search for all x,y inside the identified shape and for all distances D up to 200 bp (default).

Results of comparative testing

Figure S3. Comparison on datasets from Klepper, K., et al. 2008.

A. Average performance over all datasets.

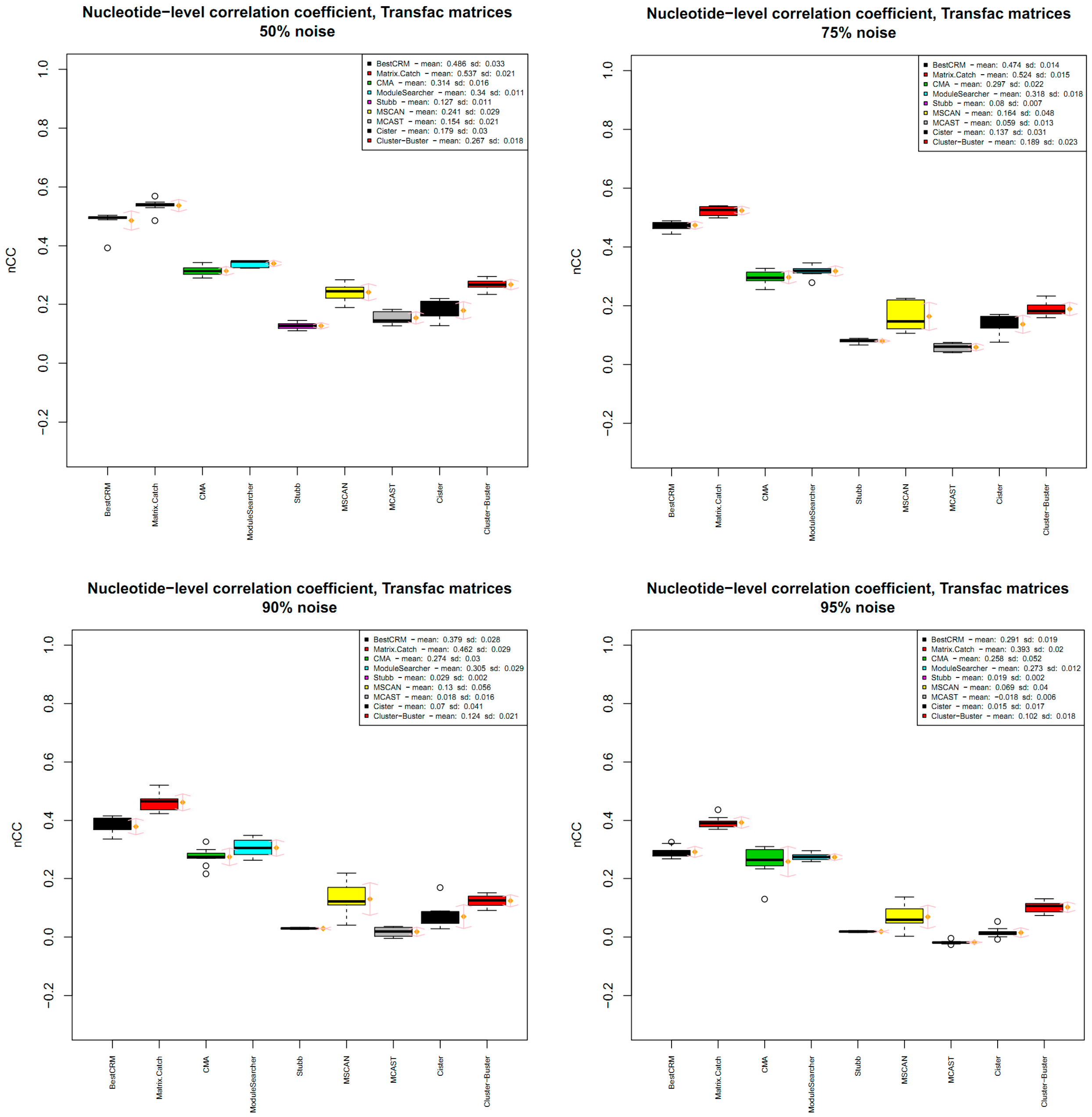

Figure S3 (Continued)**.**

B. Different performance characteristics based on nucleotide correlation coefficient (CC – first column). All performance measures have values from 0 to 1 (y-axis).

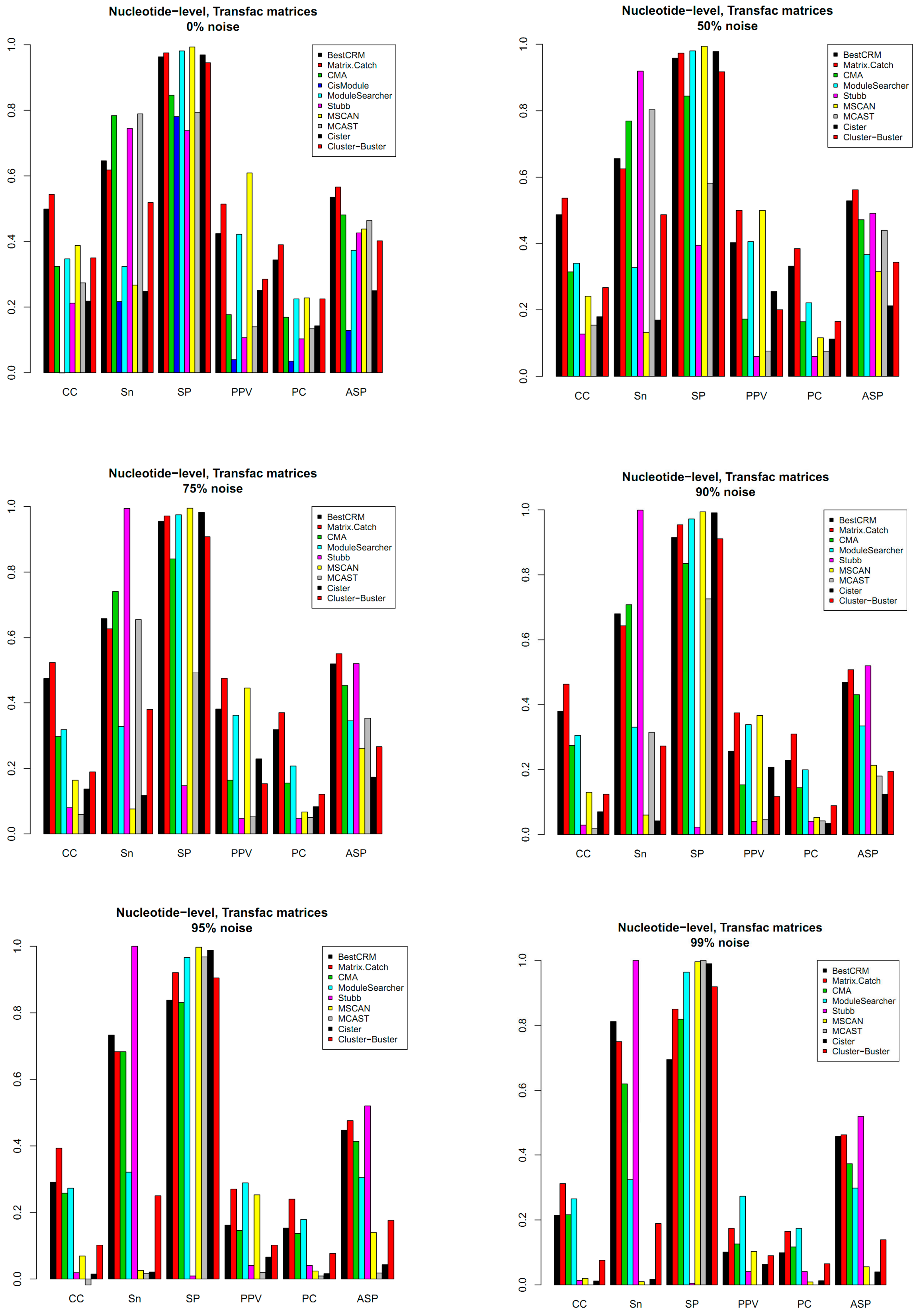

Figure S3 (Continued)**.**

C. Performance in different classes of transcription factors.

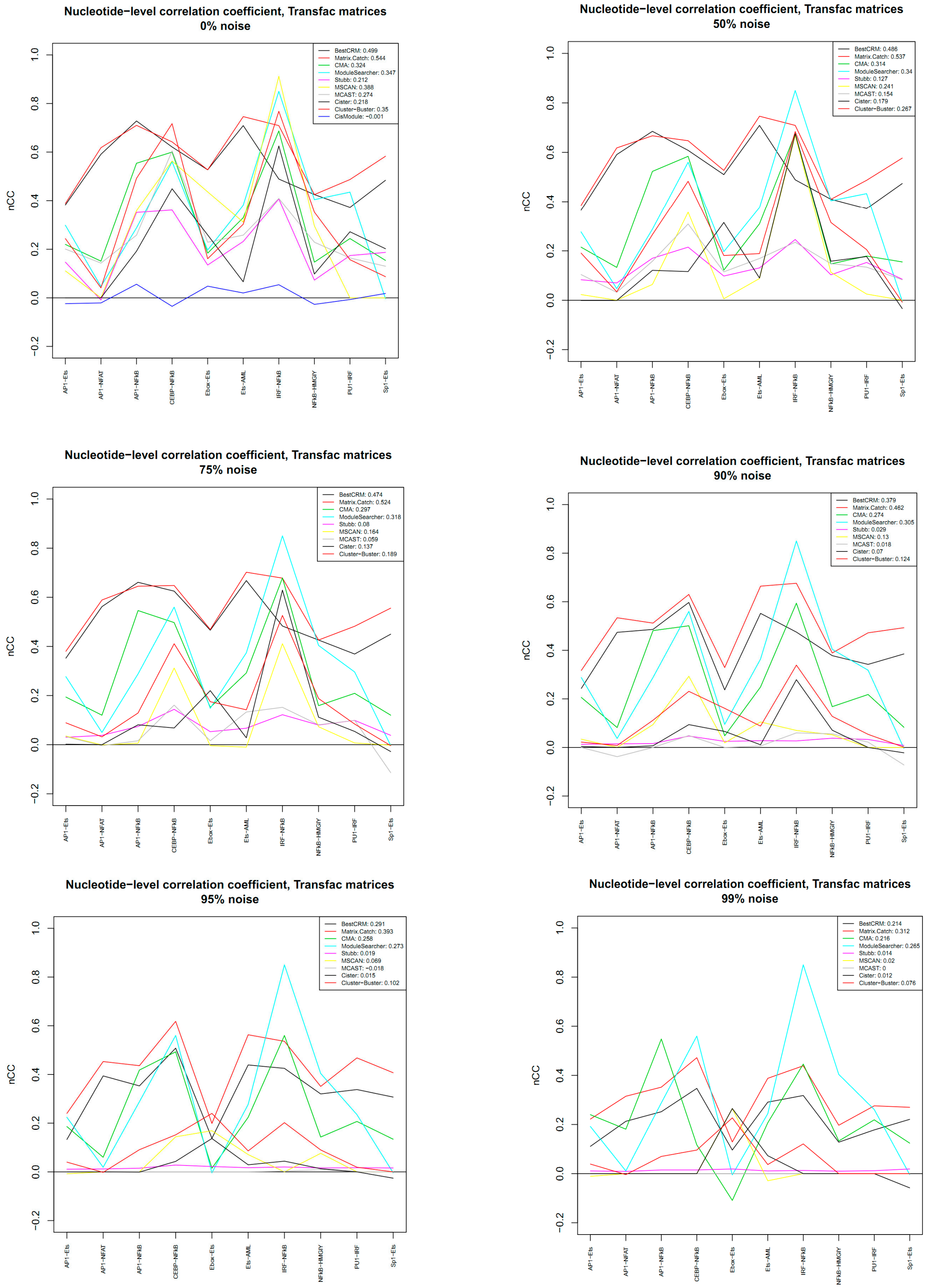

Figure S3 (Continued)**.**

D. Performance by different noise levels in datasets.

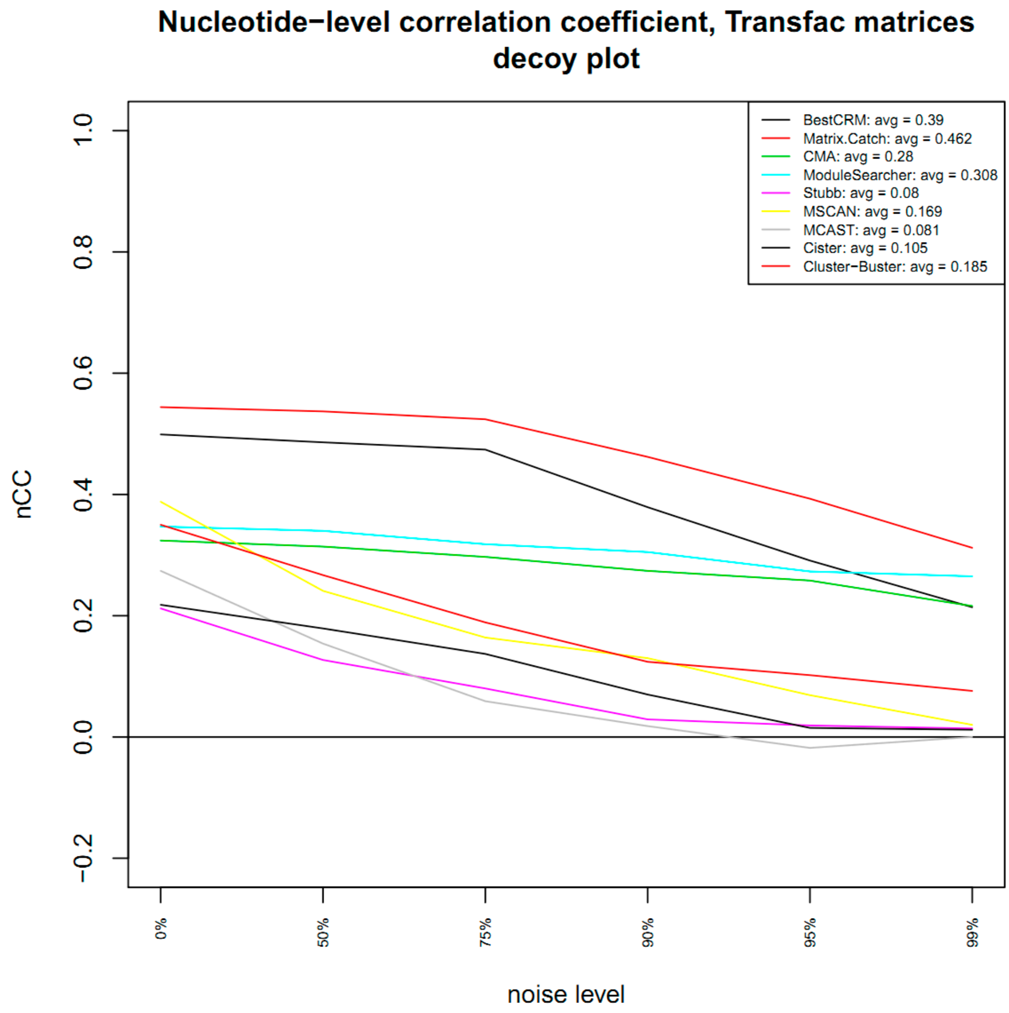

Table S1. Identification of CRMs in tissue-specific promoters by different programs. C^+ – portion of promoters with at least one predicted CRM in positive dataset, C^- – in negative dataset. #CRMs – number of different CRMs identified.

BestCRM

500bp	$C^+ \geq 0.90$ & $C^- \leq 0.50$			$C^+ \geq 0.75$ & $C^- \leq 0.50$			$C^+ \geq 0.66$ & $C^- \leq 0.50$			$C^+ \geq 0.50$ & $C^- \leq 0.25$			$C^+ \geq 0.33$ & $C^- \leq 0.15$		
	C^+	C^-	#CRMs	C^+	C^-	#CRMs	C^+	C^-	#CRMs	C^+	C^-	#CRMs	C^+	C^-	#CRMs
Breast	0.916	0.458	1	0.792	0.458	6	0.667	0.321	18	0.500	0.167	12	0.333	0.074	16
Cerebellum	-	-	-	-	-	-	-	-	-	-	-	-	-	-	-
Heart	-	-	-	0.814	0.489	1	0.676	0.386	8	0.500	0.227	1	0.349	0.141	1
Kidney	-	-	-	0.765	0.474	3	0.686	0.379	13	0.510	0.222	2	0.353	0.123	5
Liver	-	-	-	-	-	-	-	-	-	-	-	-	-	-	-
Muscle	-	-	-	-	-	-	-	-	-	-	-	-	-	-	-
Pancreas	-	-	-	0.758	0.481	2	0.672	0.479	2	-	-	-	0.397	0.146	2
Prostate	0.941	0.442	1	0.778	0.416	8	0.667	0.304	7	0.500	0.145	3	0.333	0.073	5
Spleen	-	-	-	-	-	-	0.682	0.451	2	0.512	0.236	1	0.38	0.129	2
Testes	-	-	-	-	-	-	-	-	-	-	-	-	-	-	-
Thyroid	-	-	-	0.762	0.491	1	0.662	0.470	3	-	-	-	0.376	0.134	2

1Kb	$C^+ \geq 0.90$ & $C^- \leq 0.50$			$C^+ \geq 0.75$ & $C^- \leq 0.50$			$C^+ \geq 0.66$ & $C^- \leq 0.50$			$C^+ \geq 0.50$ & $C^- \leq 0.25$			$C^+ \geq 0.33$ & $C^- \leq 0.15$		
	C^+	C^-	#CRMs	C^+	C^-	#CRMs	C^+	C^-	#CRMs	C^+	C^-	#CRMs	C^+	C^-	#CRMs
Breast	0.916	0.458	1	0.792	0.436	7	0.708	0.356	16	0.500	0.206	6	0.333	0.069	14
Cerebellum	-	-	-	-	-	-	-	-	-	-	-	-	-	-	-
Heart	-	-	-	-	-	-	0.721	0.492	8	-	-	-	0.338	0.14	1
Kidney	-	-	-	0.765	0.432	5	0.706	0.390	12	0.510	0.246	1	0.358	0.128	2
Liver	-	-	-	-	-	-	-	-	-	-	-	-	-	-	-
Muscle	-	-	-	-	-	-	-	-	-	-	-	-	-	-	-
Pancreas	-	-	-	0.758	0.462	1	0.672	0.479	2	0.597	0.196	1	-	-	-
Prostate	0.941	0.489	1	0.833	0.293	4	0.667	0.161	8	0.667	0.161	6	0.333	0.063	9
Spleen	-	-	-	-	-	-	0.663	0.492	1	-	-	-	-	-	-
Testes	-	-	-	-	-	-	-	-	-	-	-	-	-	-	-
Thyroid	-	-	-	0.757	0.486	1	0.662	0.458	4	-	-	-	0.338	0.146	1

MatrixCatch

500bp	$C^+ \geq 0.90$ & $C^- \leq 0.50$			$C^+ \geq 0.75$ & $C^- \leq 0.50$			$C^+ \geq 0.66$ & $C^- \leq 0.50$			$C^+ \geq 0.50$ & $C^- \leq 0.25$			$C^+ \geq 0.33$ & $C^- \leq 0.15$		
	C^+	C^-	#CRMs	C^+	C^-	#CRMs	C^+	C^-	#CRMs	C^+	C^-	#CRMs	C^+	C^-	#CRMs
Breast	-	-	-	0.750	0.391	17	0.667	0.279	67	0.500	0.168	20	0.333	0.063	58
Cerebellum	-	-	-	-	-	-	-	-	-	-	-	-	-	-	-
Heart	-	-	-	0.765	0.496	3	0.662	0.382	16	0.500	0.239	1	0.338	0.141	1
Kidney	-	-	-	0.765	0.479	3	0.667	0.372	43	0.529	0.235	4	0.333	0.096	17
Liver	-	-	-	-	-	-	-	-	-	-	-	-	-	-	-
Muscle	-	-	-	-	-	-	-	-	-	-	-	-	-	-	-
Pancreas	-	-	-	-	-	-	0.672	0.402	8	-	-	-	0.344	0.145	3
Prostate	0.941	0.489	1	0.765	0.305	47	0.706	0.254	74	0.529	0.071	47	0.353	0.037	80
Spleen	-	-	-	-	-	-	-	-	-	-	-	-	-	-	-
Testes	-	-	-	-	-	-	-	-	-	-	-	-	-	-	-
Thyroid	-	-	-	-	-	-	0.662	0.410	6	-	-	-	-	-	-

1Kb	$C^+ \geq 0.90$ & $C^- \leq 0.50$			$C^+ \geq 0.75$ & $C^- \leq 0.50$			$C^+ \geq 0.66$ & $C^- \leq 0.50$			$C^+ \geq 0.50$ & $C^- \leq 0.25$			$C^+ \geq 0.33$ & $C^- \leq 0.15$		
	C^+	C^-	#CRMs	C^+	C^-	#CRMs	C^+	C^-	#CRMs	C^+	C^-	#CRMs	C^+	C^-	#CRMs
Breast	-	-	-	0.750	0.359	25	0.667	0.260	54	0.500	0.167	26	0.333	0.072	51
Cerebellum	-	-	-	-	-	-	-	-	-	-	-	-	-	-	-
Heart	-	-	-	0.750	0.472	4	0.691	0.417	23	-	-	-	0.338	0.130	1
Kidney	-	-	-	0.804	0.495	5	0.667	0.368	40	-	-	-	0.333	0.101	11
Liver	-	-	-	-	-	-	-	-	-	-	-	-	-	-	-
Muscle	-	-	-	-	-	-	0.686	0.481	1	-	-	-	-	-	-
Pancreas	-	-	-	-	-	-	0.672	0.399	8	0.508	0.233	1	0.361	0.141	4
Prostate	0.941	0.442	1	0.765	0.328	47	0.706	0.252	69	0.529	0.107	39	0.412	0.056	71
Spleen	-	-	-	-	-	-	-	-	-	-	-	-	-	-	-
Testes	-	-	-	-	-	-	-	-	-	-	-	-	-	-	-
Thyroid	-	-	-	-	-	-	0.662	0.475	2	-	-	-	-	-	-

Table S1. (continued)

CMA

500bp	$C^+ \geq 0.90$ & $C^- \leq 0.50$			$C^+ \geq 0.75$ & $C^- \leq 0.50$			$C^+ \geq 0.66$ & $C^- \leq 0.50$			$C^+ \geq 0.50$ & $C^- \leq 0.25$			$C^+ \geq 0.33$ & $C^- \leq 0.15$		
	C^+	C^-	#CRMs	C^+	C^-	#CRMs	C^+	C^-	#CRMs	C^+	C^-	#CRMs	C^+	C^-	#CRMs
Breast	-	-	-	-	-	-	0.667	0.491	2	-	-	-	-	-	-
Cerebellum	-	-	-	-	-	-	-	-	-	-	-	-	-	-	-
Heart	-	-	-	-	-	-	-	-	-	-	-	-	-	-	-
Kidney	-	-	-	-	-	-	0.667	0.456	2	-	-	-	-	-	-
Liver	-	-	-	-	-	-	-	-	-	-	-	-	-	-	-
Muscle	-	-	-	-	-	-	-	-	-	-	-	-	-	-	-
Pancreas	-	-	-	-	-	-	-	-	-	-	-	-	-	-	-
Prostate	-	-	-	0.765	0.408	5	0.765	0.408	5	-	-	-	0.353	0.096	1
Spleen	-	-	-	-	-	-	-	-	-	-	-	-	-	-	-
Testes	-	-	-	-	-	-	-	-	-	-	-	-	-	-	-
Thyroid	-	-	-	-	-	-	-	-	-	-	-	-	-	-	-

1Kb	$C^+ \geq 0.90$ & $C^- \leq 0.50$			$C^+ \geq 0.75$ & $C^- \leq 0.50$			$C^+ \geq 0.66$ & $C^- \leq 0.50$			$C^+ \geq 0.50$ & $C^- \leq 0.25$			$C^+ \geq 0.33$ & $C^- \leq 0.15$		
	C^+	C^-	#CRMs	C^+	C^-	#CRMs	C^+	C^-	#CRMs	C^+	C^-	#CRMs	C^+	C^-	#CRMs
Breast	-	-	-	-	-	-	0.667	0.405	4	-	-	-	-	-	-
Cerebellum	-	-	-	-	-	-	-	-	-	-	-	-	-	-	-
Heart	-	-	-	-	-	-	-	-	-	-	-	-	-	-	-
Kidney	-	-	-	-	-	-	0.706	0.495	2	-	-	-	-	-	-
Liver	-	-	-	-	-	-	-	-	-	-	-	-	-	-	-
Muscle	-	-	-	-	-	-	-	-	-	-	-	-	-	-	-
Pancreas	-	-	-	-	-	-	-	-	-	-	-	-	-	-	-
Prostate	-	-	-	0.765	0.485	1	0.765	0.485	4	-	-	-	0.353	0.057	4
Spleen	-	-	-	-	-	-	-	-	-	-	-	-	-	-	-
Testes	-	-	-	-	-	-	-	-	-	-	-	-	-	-	-
Thyroid	-	-	-	-	-	-	-	-	-	-	-	-	-	-	-

ModuleSearcher

500bp	$C^+ \geq 0.90$ & $C^- \leq 0.50$			$C^+ \geq 0.75$ & $C^- \leq 0.50$			$C^+ \geq 0.66$ & $C^- \leq 0.50$			$C^+ \geq 0.50$ & $C^- \leq 0.25$			$C^+ \geq 0.33$ & $C^- \leq 0.15$		
	C^+	C^-	#CRMs	C^+	C^-	#CRMs	C^+	C^-	#CRMs	C^+	C^-	#CRMs	C^+	C^-	#CRMs
Breast	-	-	-	-	-	-	0.667	0.467	2	-	-	-	-	-	-
Cerebellum	-	-	-	-	-	-	-	-	-	-	-	-	-	-	-
Heart	-	-	-	-	-	-	-	-	-	-	-	-	-	-	-
Kidney	-	-	-	-	-	-	0.725	0.479	8	-	-	-	0.333	0.131	1
Liver	-	-	-	-	-	-	-	-	-	-	-	-	-	-	-
Muscle	-	-	-	-	-	-	0.674	0.499	1	-	-	-	-	-	-
Pancreas	-	-	-	-	-	-	-	-	-	-	-	-	-	-	-
Prostate	-	-	-	-	-	-	0.706	0.418	4	-	-	-	-	-	-
Spleen	-	-	-	-	-	-	-	-	-	-	-	-	-	-	-
Testes	-	-	-	-	-	-	-	-	-	-	-	-	-	-	-
Thyroid	-	-	-	-	-	-	0.662	0.498	1	-	-	-	-	-	-

1Kb	$C^+ \geq 0.90$ & $C^- \leq 0.50$			$C^+ \geq 0.75$ & $C^- \leq 0.50$			$C^+ \geq 0.66$ & $C^- \leq 0.50$			$C^+ \geq 0.50$ & $C^- \leq 0.25$			$C^+ \geq 0.33$ & $C^- \leq 0.15$		
	C^+	C^-	#CRMs	C^+	C^-	#CRMs	C^+	C^-	#CRMs	C^+	C^-	#CRMs	C^+	C^-	#CRMs
Breast	-	-	-	-	-	-	0.708	0.416	2	0.500	0.241	1.000	0.417	0.144	1
**Cerebellum**	-	-	-	-	-	-	-	-	-	-	-	-	-	-	-
Heart	-	-	-	-	-	-	0.662	0.479	1	-	-	-	-	-	-
Kidney	-	-	-	-	-	-	0.686	0.436	6	-	-	-	0.333	0.148	4
**Liver**	-	-	-	-	-	-	-	-	-	-	-	-	-	-	-
Muscle	-	-	-	-	-	-	-	-	-	-	-	-	-	-	-
Pancreas	-	-	-	-	-	-	0.672	0.471	1	-	-	-	-	-	-
Prostate	-	-	-	0.765	0.440	2	0.765	0.440	3	-	-	-	0.353	0.142	1
**Spleen**	-	-	-	-	-	-	-	-	-	-	-	-	-	-	-
**Testes**	-	-	-	-	-	-	-	-	-	-	-	-	-	-	-
Thyroid	-	-	-	-	-	-	0.662	0.474	1	-	-	-	-	-	-

Table S1. (continued)

CisModule

500bp	$C^+ \geq 0.90$ & $C^- \leq 0.50$			$C^+ \geq 0.75$ & $C^- \leq 0.50$			$C^+ \geq 0.66$ & $C^- \leq 0.50$			$C^+ \geq 0.50$ & $C^- \leq 0.25$			$C^+ \geq 0.33$ & $C^- \leq 0.15$		
	C^+	C^-	#CRMs	C^+	C^-	#CRMs	C^+	C^-	#CRMs	C^+	C^-	#CRMs	C^+	C^-	#CRMs
Breast	-	-	-	-	-	-	-	-	-	-	-	-	0.333	0.091	2
Cerebellum	-	-	-	-	-	-	-	-	-	-	-	-	-	-	-
Heart	-	-	-	-	-	-	-	-	-	-	-	-	-	-	-
Kidney	-	-	-	-	-	-	-	-	-	-	-	-	-	-	-
Liver	-	-	-	-	-	-	-	-	-	-	-	-	-	-	-
Muscle	-	-	-	-	-	-	-	-	-	-	-	-	-	-	-
Pancreas	-	-	-	-	-	-	-	-	-	-	-	-	-	-	-
Prostate	-	-	-	-	-	-	0.706	0.497	1	0.529	0.239	1	0.353	0.054	2
Spleen	-	-	-	-	-	-	-	-	-	-	-	-	-	-	-
Testes	-	-	-	-	-	-	-	-	-	-	-	-	-	-	-
Thyroid	-	-	-	-	-	-	-	-	-	-	-	-	-	-	-

1Kb	$C^+ \geq 0.90$ & $C^- \leq 0.50$			$C^+ \geq 0.75$ & $C^- \leq 0.50$			$C^+ \geq 0.66$ & $C^- \leq 0.50$			$C^+ \geq 0.50$ & $C^- \leq 0.25$			$C^+ \geq 0.33$ & $C^- \leq 0.15$		
	C^+	C^-	#CRMs	C^+	C^-	#CRMs	C^+	C^-	#CRMs	C^+	C^-	#CRMs	C^+	C^-	#CRMs
Breast	-	-	-	-	-	-	-	-	-	-	-	-	0.375	0.139	1
Cerebellum	-	-	-	-	-	-	-	-	-	-	-	-	-	-	-
Heart	-	-	-	-	-	-	-	-	-	-	-	-	-	-	-
Kidney	-	-	-	-	-	-	-	-	-	-	-	-	-	-	-
Liver	-	-	-	-	-	-	-	-	-	-	-	-	-	-	-
Muscle	-	-	-	-	-	-	-	-	-	-	-	-	-	-	-
Pancreas	-	-	-	-	-	-	-	-	-	-	-	-	-	-	-
Prostate	-	-	-	-	-	-	-	-	-	-	-	-	-	-	-
Spleen	-	-	-	-	-	-	-	-	-	-	-	-	-	-	-
Testes	-	-	-	-	-	-	-	-	-	-	-	-	-	-	-
Thyroid	-	-	-	-	-	-	-	-	-	-	-	-	-	-	-

Figure S4. The problem of similar PWMs.

Formally different PWMs in libraries, can represent very similar motifs (including its reverse complements). The problem is exemplified with application of PC-TraFF to breast dataset. Top 5 identified CRMs are presented.

PC-TraFF Significant Pairs

Pairs	Z-Score	Reference
V$PU1_Q6 - V$ETS_Q6 (Info)	8.96628	TRANSCompel, BioGRID
V$ETS_Q4 - V$ETS_Q6 (Info)	5.59389	TRANSCompel, BioGRID
V$STAT6_01 - V$CEBP_Q2_01 (Info)	5.13913	TRANSCompel, BioGRID
V$STAT6_01 - V$OCT_Q6 (Info)	4.94159	
V$AP1_Q2_01 - V$AP1_Q4_01 (Info)	4.64285	TRANSCompel, BioGRID
V$AP1_C - V$AP1_Q2_01 (Info)	4.53015	TRANSCompel, BioGRID
V$SP1_Q6_01 - V$SP1_Q2_01 (Info)	4.32263	TRANSCompel, BioGRID
V$OCT_Q6 - V$FOX_Q2 (Info)	4.28302	
V$AP1_01 - V$OCT_Q6 (Info)	3.84685	TRANSCompel
V$OCT_Q6 - V$CEBP_Q2_01 (Info)	3.82711	BioGRID
V$CETS1P54_01 - V$SP1_Q6_01 (Info)	3.78187	TRANSCompel
V$SF1_Q6 - V$ETS_Q6 (Info)	3.69834	
V$CEBP_Q2_01 - V$AP1_Q4_01 (Info)	3.66405	TRANSCompel, BioGRID
V$AP1_Q6 - V$AP1_Q4_01 (Info)	3.65580	TRANSCompel, BioGRID
V$SOX9_B1 - V$STAT6_01 (Info)	3.65478	
V$GR_Q6_01 - V$PR_Q2 (Info)	3.63254	TRANSCompel, BioGRID
V$MYB_Q6 - V$MYB_Q3 (Info)	3.59763	
V$EGR_Q6 - V$SP1_Q6_01 (Info)	3.58168	BioGRID
V$CEBPB_01 - V$STAT6_01 (Info)	3.57294	TRANSCompel

CRM1:

Pair Information

PWM	Sequence Logo	Linked TFs
V$PU1_Q6	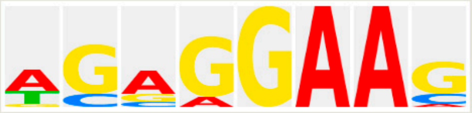	SPI1
V$ETS_Q6	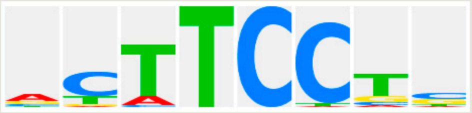	ELF1, ELF2, ELF4, ELK1, ELK3, ELK4, ERG, ETS1, ETS2,

CRM2:

Pair Information		
PWM	Sequence Logo	Linked TFs
V$ETS_Q4	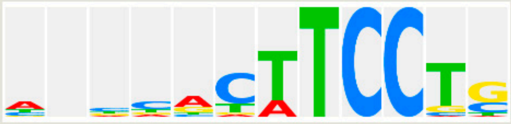	ELF1, ELF2, ELK1, ELK4, ERF, ERG, ETS1, ETS2, ETV7, FLI1
V$ETS_Q6	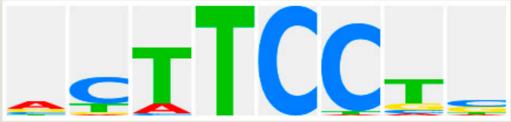	ELF1, ELF2, ELF4, ELK1, ELK3, ELK4, ERG, ETS1, ETS2,

CRM3:

Pair Information		
PWM	Sequence Logo	Linked TFs
V$STAT6_01	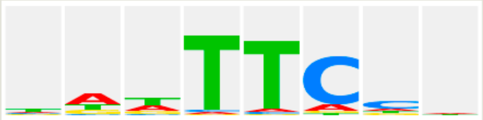	STAT6
V$CEBP_Q2_01	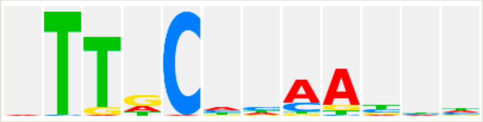	CEBPA, CEBPB, CEBPD, CEBPE, CEBPG

CRM4:

Pair Information		
PWM	Sequence Logo	Linked TFs
V$STAT6_01	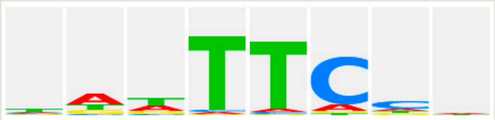	STAT6
V$OCT_Q6	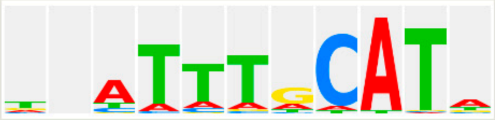	POU2AF1, POU2F1, POU2F2, POU2F3, POU3F1, POU3F2, POU3F3, POU4F1

CRM5

Pair Information		
**PWM**	**Sequence Logo**	**Linked TFs**
V$AP1_Q2_01	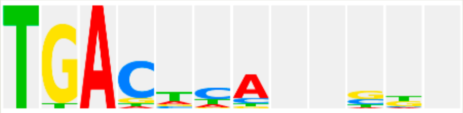	FOS, FOSB, FOSL1, FOSL2, JUN
V$AP1_Q4_01	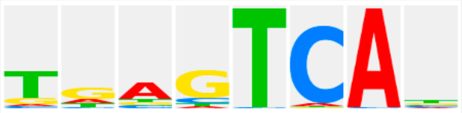	FOS, FOSB, FOSL1, FOSL2, JUN
